# Contact pathway in surgical and transcatheter aortic valve replacement

**DOI:** 10.3389/fcvm.2022.887664

**Published:** 2022-07-22

**Authors:** María Eugenia de la Morena-Barrio, Javier Corral, Cecilia López-García, Víctor Alonso Jiménez-Díaz, Antonia Miñano, Pablo Juan-Salvadores, María Asunción Esteve-Pastor, José Antonio Baz-Alonso, Ana María Rubio, Francisco Sarabia-Tirado, Miguel García-Navarro, Juan García-Lara, Francisco Marín, Vicente Vicente, Eduardo Pinar, Sergio José Cánovas, Gonzalo de la Morena

**Affiliations:** ^1^Centro Regional de Hemodonación, Servicio de Hematología y Oncología Médica, Hospital Universitario Morales Meseguer, IMIB-Arrixaca, Centro Investigacion Biomédica en red Enferemedades Raras (CIBERER), CEIR Campus Mare Nostrum (CMN), Universidad de Murcia, Murcia, Spain; ^2^Servicio de Cardiología, Hospital Clínico Universitario Virgen de la Arrixaca, Universidad de Murcia, IMIB-Arrixaca, Centro de Investigación Biomédica en Red Enfermedades Cardiovasculares (CIBERCV), Murcia, Spain; ^3^Unidad de Investigación Cardiovascular, Servicio de Cardiología, Hospital Álvaro Cunqueiro, Vigo, Spain; ^4^Servicio de Radiología, Hospital Clínico Universitario Virgen de la Arrixaca, Murcia, Spain; ^5^Servicio de Cirugía Cardiovascular, Hospital Clínico Universitario Virgen de la Arrixaca, Murcia, Spain

**Keywords:** contact pathway, aortic valve replacement, thrombosis, factor XI, factor XII, kallikrein

## Abstract

**Background:**

Aortic valve replacement is the gold standard treatment for severe symptomatic aortic stenosis, but thrombosis of bioprosthetic valves (PVT) remains a concern.

**Objective:**

To analyze the factors involved in the contact pathway during aortic valve replacement and to assess their impact on the development of thromboembolic complications.

**Methods:**

The study was conducted in 232 consecutive patients who underwent: transcatheter aortic valve replacement (TAVR, *N* = 155), and surgical valve replacement (SAVR, *N* = 77) (MUVITAVI project). Demographic and clinical data, outcomes including a combined end point (CEP) of thrombotic events, and imaging controls were recruited. Samples were collected 24 h before and 48 h after valve replacement. FXII, FXI and (pre)kallikrein were evaluated by Western Blot and specific ELISA with nanobodies.

**Results:**

The CEP of thrombotic events was reached by 19 patients: 13 patients presented systemic embolic events and 6 patients subclinical PVT. Valve replacement did not cause FXII activation or generation of kallikrein. There was a significant reduction of FXI levels associated with the procedure, which was statistically more pronounced in SAVR than in TAVR. Cases with reductions of FXI below 80% of basal values had a lower incidence of embolic events during the procedure than patients in whom FXI increased above 150%: 2.7 vs. 16.7%; *p*: 0.04.

**Conclusion:**

TAVR or SAVR did not significantly activate the contact pathway. A significant reduction of FXI, was observed, particularly in SAVR, associated with lower incidence of thrombotic events. These results encourage evaluating the usefulness and safety of FXI-directed antithrombotic treatments in these patients.

## Introduction

Aortic stenosis (AS) has become the most common primary heart valve disease and an major cause of cardiovascular morbidity and mortality (1). Aortic valve replacement is the treatment of choice for symptomatic patient who undergo more than > 100.000 procedures annually in the United States alone (2). Bioprosthetic valves are by far the most commonly implanted valves, representing almost 80% of surgical aortic valve replacements (SAVR) (3) and all implanted transcatheter valves (TAVR). In recent years, different studies have warned about the development of bioprosthetic valves thrombosis (PVT). Clinical or subclinical thrombosis has been described in up to 10% of the patients and this complication seems to be associated with higher frequency of prosthetic dysfunction, embolic events and the need for replacement (4, 5). The significant morbidity and mortality associated with this condition warrants rapid diagnostic evaluation and predictive tools. However, diagnosis can be challenging, mainly because of variable clinical presentations, the diagnostic tools used, and the degree of valvular obstruction.

In this framework, it is interesting to analyze the impact of aortic valve replacement in different systems, particularly those involved in thrombosis, the most frequent complication observed in these patients. These studies could identify new biomarkers and new treatment targets that could improve the outcome of valve replacement. The haemostatic system is an excellent candidate to be evaluated. Particularly interesting is the contact pathway of coagulation, as it has minor physiological relevance but is activated by negatively charged surfaces and artificial surfaces of medical devices (6). The contact pathway includes three elements with proteolytic activity: factor XII (FXII), factor XI (FXI) and (pre)kallikrein, which are involved in multiple vital proteolytic cascades: host defense, inflammation and coagulation after activation by negatively charged surfaces (7). Although deficiency or inhibition of these elements has no or minor physiological consequences on hemostasis (8), recent evidence support a key pathological role for these contact elements, and different approaches are being developed to target FXII, FXI, or (pre)kallikrein with antithrombotic potential and minor risk of bleeding (9, 10). Indeed, the elements of the contact pathway have been suggested as potential targets of new anticoagulant treatments for patients receiving medical devices (11–13). This is a relevant issue because antithrombotic therapy after aortic valve replacement has not yet been defined (14).

**FIGURE 1:**
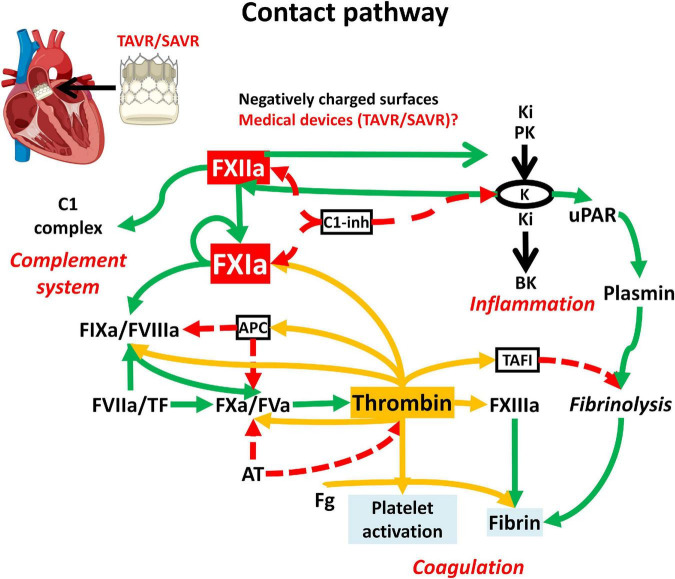
Scheme of the contact pathway and its involvement in coagulation, inflammation and complement system. Surgical (SAVR) or transcatheter (TAVR) aortic valve is illustrated as a potential trigger of FXII autoactivation. Dashed red arrows indicate inhibition; green arrows activation; yellow arrows activation by thrombin. APC, activated protein C; Ki, Kininogen; PK, prekallikrein; K, kallikrein; BK, bradykinin; TAFI, thrombin activable fibrinolysis inhibitor; Fg, fibrinogen; C1-inh, C1-inhibitor; uPAR, urokinase-type plasminogen; TF, Tissue Factor.

Our study has been designed to explore the impact of aortic valve replacement on the contact pathway elements with the aim of finding new biomarkers useful for the management of these patients as well as new clues for the best antithrombotic treatment in this scenario (Figure 1).

## Patients and methods

### Patients

The MUVITAVI (**MU**rcia and **VI**go **T**ranscatheter **A**ortic **V**alvular **I**mplantation) project is an observational study of patients who underwent two aortic valve replacement procedures (TAVR and SAVR) consecutively recruited between February-2018 to February 2020 from two Spanish referenced hospitals: Hospital Clínico Universitario Virgen de la Arrixaca in Murcia, and Álvaro Cunqueiro in Vigo.

The inclusion criteria were age over 50 years, and to give their informed consent to enter the study, which was approved by the Ethics Committees from each Hospital and performed in accordance with the 1964 Declaration of Helsinki and their later amendments.

Patients with single and programmed procedures were included. Exclusion criteria included: emergency or urgent procedures two or more valves treated any suspicion of endocarditis or combined coronary revascularization.

### Clinical data, imaging controls, and follow-up

Demographics and clinical data, comorbidities, and outcomes were recruited by independently observers blinded to hematologic analysis. Patients were followed-up every 6 months. Periprocedural complications, clinical follow-up, transthoracic echocardiography (TTE) and computer tomography imaging (CT) results were incorporated using an online standardized data collection.

### Sample collection

Samples were collected 24 h before and 48 h after the replacement in vacuum tubes containing 0.109 M sodium citrate. Platelet-poor plasma (PPP) was obtained within 2 h after extraction by centrifugation (2000 *g* × 15 min) at 20°C and then stored at -80°C.

As reference plasma we used a pool of 100 healthy blood donors. Moreover, as controls we also included plasma of one patient with congenital FXI deficiency caused by the p.Cys416Tyr pathogenic gene variation in heterozygosis (FXI:C 37%), and plasma of another patient with congenital FXII deficiency caused by a homozygous deletion of a single nucleotide (c.919del G) causing a frameshift (p.Val307Cysfr*44) (FXII:C 3%).

Variations of FXI levels were calculated as the ratio between FXI levels after 48 h from procedure (B) and the FXI basal levels (A), and expressed in percentages, FXI (B/A) (%).

### Contact pathway analysis

Plasma FXII, FXI and (pre)kallikrein were evaluated by SDS-PAGE and Western Blot using procedures described elsewhere (15, 16). Two antibodies targeting FXI were used: GAFXI-AP (Enzyme Research, United Kingdom) detecting the dimer under not-reducing conditions and GAFXI-HRP (Enzyme Research, United Kingdom) that also detects FXIa under reducing conditions using dithiothreitol. Densitometry analysis of Western blot results was done with ImageJ (17).

Quantification of FXIIa-C1 inhibitor and FXIa-C1 inhibitor complexes were done with specific ELISA using nanobodies provided by ISTH Cosyne programme (18, 19). Values were represented as percentages of those observed in kaolin-full activated reference plasma.

Determination of FXI levels was also done with a functional assay using the FXIa specific chromogenic substrate SC2366 after full activation of the contact pathway with kaolin.

### Procoagulant markers

Activation of the coagulation cascade was evaluated by determination of antithrombin activity using a functional anti-FXa assay with heparin, bovine FXa, and S-2765 chromogenic substrate (HemosIL TH, Instrumentation Laboratory, Italy) and by quantifying thrombin-antithrombin (TAT) complexes by SDS-PAGE Western blot under reducing conditions using a polyclonal antibody (A9522, Sigma-Aldrich) and by a specific ELISA [ab108907 –Thrombin-Antithrombin Complex (TAT) Human ELISA Kit, Abcam].

### Genetic analysis

The exons and flanking regions of *F12* gene were amplified and sequenced as described before (20).

### Thrombotic events

We recruited all embolic events happened during follow-up period. A composite end point (CEP) of thrombotic events included: any peripheral embolic event, transient ischaemic attack or stroke, and clinical or subclinical PVT. Diagnosis of subclinical PVT required at least one of the three next imaging criteria: a twofold increase in mean transaortic Doppler gradient, mobile thrombus attached to the prosthesis, or the presence of hypo attenuated leaflet thickening (HALT) with reduced leaflet motion on CT (5).

### Statistical analysis

Descriptive analysis of qualitative variables included percentages. Normally distributed continuous variables were presented as means ± standard deviations (SD), whereas non-normally distributed variables were presented as median and interquartile ranges (IQR). Pearson’s Chi-Squared test and Fisher’s exact test were used for comparison of proportions or ordinal variables. Kolmogorov–Smirnov and Shapiro–Wilk tests were used for testing normality of continuous variables. Student’s-T (parametric) or Mann–Whitney-U tests were used for comparison of two means and analysis of variance (ANOVA, parametric) or Kruskal–Wallis (non-parametric) tests were used for comparison of more than two means. Apart from *p*-values, 95% Confidence Intervals (95% CI) were also calculated.

Statistical analysis was performed with the use of Excel^^®^^ (Microsoft), GraphPad Prism^^®^^ (GraphPad Software), and IBM SPSS Statistics 21^^®^^ (IBM SPSS Software).

## Results

### Characteristics of the cohort

A total of 232 patients were included: 155 in TAVR and 77 in SAVR group. Table 1 shows the main characteristics of the patients.

**TABLE 1:** Demographic, clinical characteristics, and clinical outcomes of patients underwent TAVR or SAVR.

	Total N: 232	TAVR N: 155	SAVR N: 77	*p*
Sex male (%)	103 (44.4)	66 (42.6)	37 (48.1)	NS
Age (years) (IQR)	81 (75–84)	83 (80–85)	75 (72–77)	0.000
EuroScore II (IQR)	2.08 (1.36–3.35)	2.73 (1.84–4.23)	1.34 (1.07–1.81)	0.000
STS score	2.45 (1.59–3.84)	3.05 (2.27–4.65)	1.59 (1.13–2.09)	0.000
Medical history				
Diabetes Mellitus (%)	110 (47.8)	80 (52.3)	30 (27.3)	0.03
Hypertension (%)	203 (87.5)	136 (87.7)	67 (87.0)	NS
Chronical Kidney Disease (%)	47 (20.3)	38 (24.7)	9 (11.7)	0.01
Ischemic Heart Disease (%)	70 (30.2)	53 (34.2)	17 (22.1)	0.03
Myocardial Infarction (%)	24 (10.3)	17 (11.0)	7 (9.1)	NS
Stroke (%)	27 (11.6)	19 (12.6)	8 (10.3)	NS
Atrial Fibrillation (%)	62 (26.7)	49 (31.6)	13 (16.9)	0.01
Antiplatelet therapy (%)	99 (42.9)	70 (45.2)	29 (38.2)	NS
Anticoagulant				
AVK (%)	42 (18.2)	33 (21.3)	9 (11.8)	NS
DOAC (%)	13 (5.6)	13 (8.4)	0 (0)	0.00
Echocardiography				
Mean transvalvular aortic gradient (mmHg) (IQR)	44.0 (37.5–53.4)	43.5 (37.5–53.0)	48.0 (37.2–54.0)	NS
AVA (cm^2^)	0.72 ± 0.19	0.72 ± 0.20	0.71 ± 0.18	NS
LVEF (%) (IQR)	60 (53.6–66.0)	60.0 (52.6–65.9)	61.0 (55.0–66.4)	NS
Clinical outcome at 2 years of follow-up				
	**N: 229**	**N: 154**	**N: 75**	
CVD, N (%)	7 (3.1)	6 (3.9)	1 (1.3)	NS
Non-CVD, N (%)	15 (6.6)	13 (8.4)	2 (2.7)	NS
All cause death, N (%)	22 (9.6)	19 (12.3)	3 (4.0)	0.048
Subclinical PVT, N (%)	6 (2.6)	5 (3.2)	1 (1.3)	NS
Embolic Event, N (%)	13 (5.6)	10 (6.5)	3 (4.0)	NS
CEP, N (%)	19 (8.3)	15 (9.7)	4 (5.3)	NS

AVA, aortic valve area; AVK, antivitamin K; DOAC, direct oral anticoagulants; LVEF, left ventricular ejection fraction; TAVR, transaortic valve replacement; SAVR, surgical aortic valve replacement. CEP, Combined end point; CVD, cardiovascular deaths; PVT, prosthetic valve thrombosis. Values are n (%), mean ± SD, or median (IQR). NS, No Significant, p > 0.05.

Patients in the TAVR group were older and had higher mortality risk scores and comorbidity burden than patients in the SAVR group. Likewise, the incidence of atrial fibrillation and the use of direct oral anticoagulants were significantly higher in the TAVR group. Echocardiographic parameters were comparable between both groups.

In SAVR group, 39 patients (50.6%) had a “sutureless” type of rapid deployment prosthesis: 30 Perceval^^®^^, and 9 Edwards Intuity^^®^^; while 38 (49.3%) received a “sutured” type prosthesis: 22 Edwards Perimount Magna Ease^^®^^, 10 Crown PRT^^®^^, 3 Trifecta SJM^^®^^, 2 Edwards Inspiris^^®^^ and 1 Carbomedics Top Hat^^®^^. In TAVR group, 106 patients (68.4%) had a balloon-expandable prosthesis Edwards Sapiens^^®^^, and 49 (31.6%) a self-expandable valve: 29 Core Valve^^®^^, 16 Allegra^TM ^®^^, 2 Acurate^^®^^, and 2 Portico^^®^^.

After the procedure all patients were on standardized treatment with double antiplatelet therapy (aspirin and clopidogrel). Additionally, 49 (31%) patients in TAVR group and 7 (9.4%) patients in SAVR group were anticoagulated with either antivitamin K or direct oral anticoagulant.

### Activation of the contact pathway

**FIGURE 2:**
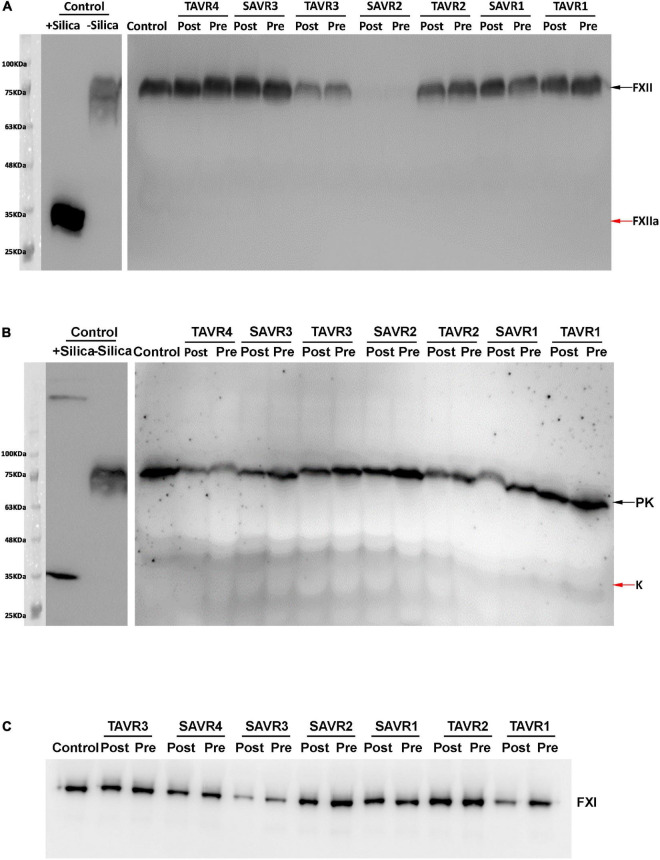
Plasma FXII **(A)**, Pre(Kallikrein) **(B)**, and FXI **(C)** detected by Western Blot after SDS-PAGE in representative samples of patients underwent TAVR or SAVR. The samples pre and post-procedure are indicated. Zymogens are pointed by black arrows, while activated forms are pointed by red arrows. As control of activation, plasma from a pool of 100 healthy blood donors was treated with silica.

We firstly evaluated a potential activation of the contact pathway by the replacement of a cardiac valve. Our results show that, regardless of the procedure, it did not cause detectable activation of FXII by Western Blot (Figure 2A). Consistent with this result, neither TAVR nor SAVR caused generation of detectable kallikrein in plasma by Western Blot (Figure 2B).

**FIGURE 3:**
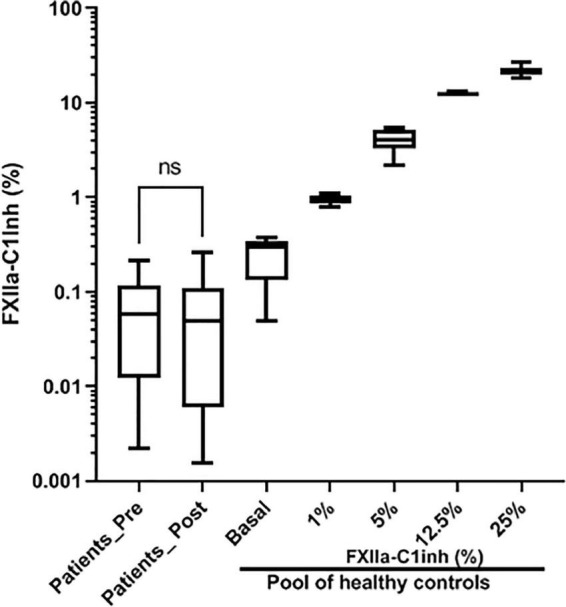
Levels of FXIIa-C1 Inhibitor complexes in pre- and post-procedure samples. Values, referenced as percentages of kaolin-full activated reference pool plasma generated 100 healthy subjects, were determined by a specific ELISA using nanobodies. ns: *p* > 0.05.

Quantification of FXIIa-C1 inhibitor complexes by a specific ELISA using nanobodies revealed negligible activation of FXII either in basal samples or in samples collected after the transcatheter aortic valve replacement process (Figure 3), independently of the replacement procedure (TAVR; pre = 0.069 ± 0.066% post = 0.077 ± 0.065%; or SAVR; pre = 0.049 ± 0.044% post = 0.024 ± 0.038%).

**FIGURE 4:**
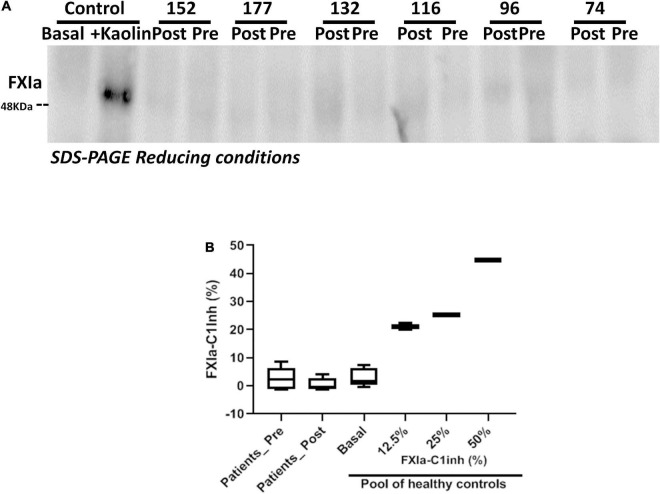
FXIa levels in patient samples pre and post-procedure. **(A)** Representative Western blot of plasma samples of patients pre- and post- procedure. As control, plasma from a healthy subject was also evaluated without and after full activation with kaolin; **(B)** FXIa-C1 inhibitor complexes quantified by ELISA using nanobodies. As standards, a basal sample, and dilutions of fully kaolin-activated plasma of a pool of 100 healthy control subjects were used.

FXI activation, assessed by two methods, Western blot analysis using reducing conditions and by a FXIa-C1 inhibitor specific ELISA using nanobodies, revealed negligible FXI activation either in basal samples or in samples collected post-procedure (Figure 4), regardless of the replacement procedure [TAVR; pre = 0.03(0.01–0.14)% post = 0.10(0.01–0.67)%; or SAVR; pre = 3.49(0.01–7.04)% post = 0.27(0.02–3.27)].

### Variations of FXII and FXI levels

Significant variations in FXII levels were observed between patients (Figure 2A), but as expected, they correlated with the functional and common polymorphism affecting the Kozak sequence of *F12*.

One patient, TAVR44, an 87-year-old-male patient who received TAVR, had a complete FXII deficiency, similar to that found in a patient with congenital FXII deficiency (Supplementary Figure 1A). As expected, all other liver proteins tested (antithrombin and FXI) reached normal levels, similar to that found in the congenital FXII-deficient patient (Supplementary Figure 1B). Genetic analysis of *F12* revealed a new missense mutation, not found in ExAC or 1000 Genomes, that located in exon 11: c.1277 T > G; p.Val426Gly Supplementary Figure 1C). This genetic variant, which was found in heterozygous state, was a disease-causing mutation as predicted by Mutation Taster (21). Additionally, the patient was homozygous for an intronic variant that might affect the correct splicing of this gene: c.1251 –9^°^C > T, a frequent gene variation (MAF: 0.6517) with benign prediction (Supplementary Figure 1C) and homozygous T/T for the functional Kozak polymorphism (22). TAVR44 had hypertension, Charlson comorbidity index of 4, STS score of 3.36, EuroScore II of 1.94, severe aortic valve stenosis with mild insufficiency, angina and dyspnea, NYHA III, and atrial fibrillation. The patient was under oral anticoagulation (Rivaroxaban 15 mg). The patient underwent transfemoral TAVR (Edwards Sapiens N°29). No complications were recorded during the procedure. During the 6-month follow-up no adverse event was observed. FXI levels in this patient were normal according to Western blot and functional assays, and no activation of FXI was observed by Western blot or ELISA either in both samples, basal and post-TAVR procedure (Supplementary Figure 1D).

Finally, no differences in antithrombin activity were detected in basal and post-procedure samples (85 and 92%, respectively) and no TAT complexes were observed in baseline or post-procedure samples (Supplementary Figure 1B).

For the entire cohort, no significant differences were observed in the levels of FXII and FXIIa-C1 inhibitor in the samples collected before and 2 days after the valvular replacement procedure (Figures 2, 3).

**FIGURE 5:**
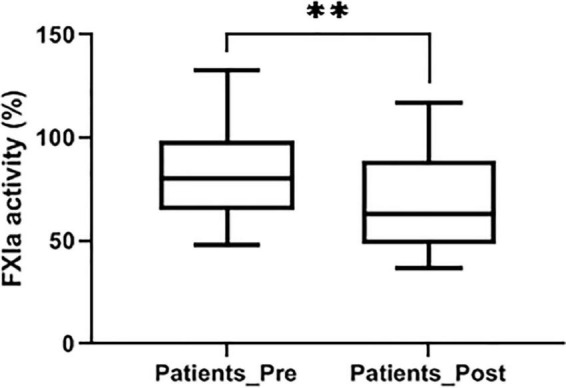
FXI levels determined by a chromogenic method after full activation with kaolin in pre- and post-procedure samples. Values were represented as% of a reference pool of plasma from 100 healthy subjects. **p < 0.01.

No patient included in this study had FXI or (pre)kallikrein deficiency (Figures 2B,C). As described, FXI levels showed an interpersonal heterogeneity (Figure 2C). Interestingly, in 60.8% of patients the replacement of the cardiac valve reduced FXI levels in the post-procedure sample compared to the levels observed in the pre-procedure sample. Only 18 patients showed relevant increases of FXI levels (> 150% of the basal levels) after the procedure (7.8%), but in 75 cases (32.3%) FXI levels in the post-procedure sample were lower than 80% of the value observed in the baseline sample (Figure 2C). Functional assays confirmed the reduction of FXI levels in post-procedure samples (Figure 5).

The type of procedure showed a significant effect in reducing FXI levels after the valve replacement. Thus, patients who underwent SAVR showed a more severe reduction in FXI levels than TAVR [86.0% IQR (67.0–108.5) vs. 94.5% IQR (78.7–110.0); *p* = 0.035]. The percentage of patients with significant reduction of FXI levels in the post-procedure sample (< 80%) was also higher among SAVR patients than TAVR patients (35/78: 44.9% vs. 40/154: 26.0%, respectively; *p* = 0.004).

The type of bioprosthesis, balloon-expandable vs. self-expandable, in TAVR group (100.5 ± 42.9 vs. 100.0 ± 35.3; *p* = ns), or sutureless vs. sutured (98.1 ± 44.8 vs. 85.4 ± 29.7; *p*: 0.06) in SAVR patients, did not affect FXI levels during the procedure.

All-cause death at 30-day follow-up was 1.3% without differences between groups: 1 patient on TAVR and 2 patients on SAVR. Nineteen patients had bleeding requiring transfusion: 13 (8.4%) TAVR, 6 (7.8%), SAVR (*p* = ns) and 24 patients required permanent pacemaker: 23 (14.5%) TAVR and 1 (1.3%) SAVR (*p* = 0.001).

Clinical follow-up was completed in all 229 patients who survived the first month. Table 1 shows clinical complications during a period of 22.1 ± 9.9 months (median 24 months).

Thromboembolic events after TAVR or SAVR were distributed as follows: 13 patients presented with systemic embolic events (10 stroke and 3 peripheral embolism), and only 6 patients had subclinical PVT. So, the CEP of thrombotic events was reached by 19 patients without differences between groups.

We analyzed in detail the potential role of FXI variations in the complications of these procedures. Patients with thrombotic complications or periprocedural mortality showed increased FXI levels in samples collected post-procedure compared to those from the pre-procedure, although results did not show statistical significance (96.2 ± 37.3 vs. 116.0 ± 43.1, *p* = 0.086, Supplementary Figure 2). Moreover, patients with elevated FXI levels after the procedure (> 150% of baseline values) showed a higher incidence of thrombotic events or periprocedural mortality than that found in patients with reduced FXI [4/18 (22.2%) vs. 2/75 (2.7%); *p* = 0.02] (Table 2). Among those who showed reductions in the FXI levels after the procedure, only 2 out of 75 (2.7%) had embolic events and no one died during the procedure.

**TABLE 2:** Complications described in patients with cardiac valve replacement according to the evolution of FXI levels after the procedure.

FXI (B/A) (%)	Group	N	CEP	CEP and PPD	CEP and CVD	Bleeding
< 80%	SAVR	40	2	2	4	4
	TAVR	35	0	0	1	3
	TOTAL	75	2 (2.7%)	2 (2.7%)	5 (6.7%)	7 (9.3%)
> 150%	SAVR	7	2	3	3	0
	TAVR	11	1	1	1	1
	TOTAL	18	3 (16.7%)	4 (22.2%)	4 (22.2%)	1 (5.6%)
**P**			0.048	0.02	0.067	0.608

CEP, Combined end point of thrombotic events, PPD, periprocedural dead; CVD, cardiovascular dead. FXI (B/A) (%): (levels of FXI 48 h after procedure/FXI basal levels) × 100.

On the other hand, 7 patients with reduced FXI after the cardiac valve replacement suffered severe bleeding during the procedure that required transfusions (9.3%) (Table 2). The incidence of bleeding requiring transfusion was similar in both groups (Table 2).

**FIGURE 6:**
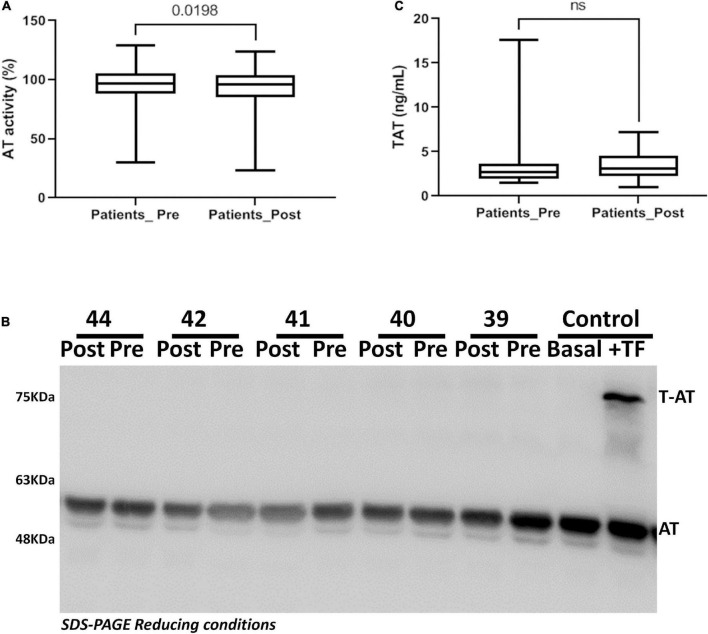
Antithrombin activity in patients underwent transcatheter aortic valve replacement. **(A)** Anti-FXa activity. **(B)** Thrombin-Antithrombin complexes quantified by ELISA. **(C)** Plasma antithrombin detected by Western blot. Representative samples are shown.

Coagulation activation was studied by quantifying two procoagulant markers: antithrombin levels by a functional method, and TAT by ELISA. As shown in Figure 6, independently of the procedure, transcatheter valve replacement caused minor, if any, activation of the coagulation cascade.

The influence of anticoagulation on the variation of FXI level was also evaluated. 42/232 patients (18%) were anticoagulated with antivitamin K before the procedure. The ratio pre/post of FXI levels was not associated with anticoagulation (anticoagulated: 104 ± 44 vs. not anticoagulated: 95 ± 36, *p* = 0.113).

Finally, we have evaluated the impact on FXI of possible confounder factors such as renal and hepatic function, left ventricular ejection fraction, pulmonary hypertension and other intraoperative and post-operative complications (pericardial drainage, permanent pacemaker, severe hemorrhage, cardiac tamponade, atrial fibrillation, infections, pleural effusion), and we found no significant associations (data not shown).

## Discussion

This is the first study to evaluate the contact pathway in a large cohort of consecutive patients who underwent aortic valve replacement, TAVR or SAVR. Our results showed that these two procedures do not activate the contact pathway. Moreover, post-procedural reduction of FXI levels was associated with a lower incidence of thrombotic events, particularly in SAVR.

Recent evidence suggests an emerging relevance for the contact pathway in medicine. First, the number of disorders involving elements of the contact pathway is increasing due to the key role of the proteases of this pathway in different systems, such as inflammation, coagulation, immune response and fibrinolysis (7, 23). Moreover, the role of FXI, FXII and (pre)kallikrein in hemostasis and thrombosis has changed dramatically. FXI deficiency used to be considered a congenital coagulopathy (Hemophilia C), but it is now widely accepted that patients with FXI deficiency have minor risk of bleeding (24). In contrast, very recent epidemiological data support that FXI deficiency strongly protects against cardiovascular and venous thromboembolism events (25). Animal models support that deficiency of FXI or FXII, or the inhibition of these proteases by different compounds strongly protect against venous or arterial thrombosis without risk of bleeding (9), and a clinical trial using anti-FXI oligonucleotides demonstrates a better antithrombotic efficacy and lower risk of bleeding than classical anticoagulants (heparin) (26). On the other side, high levels of FXI have been shown to be a strong risk factor for both venous and arterial thrombosis (27, 28) and with an increased risk of incident ischemic stroke but not hemorrhagic stroke or incident acute coronary syndrome (29).

Moreover, activation of this pathway has been involved in different disorders with an inflammatory or coagulant phenotype, such as hereditary angioedema or sepsis (30, 31). Although some molecules have been proposed as activators of the contact pathway, such as pathogen surfaces, platelet polyphosphates, subendothelial collagen, misfolded proteins, neutrophils, glycosaminoglycans, or nucleic acids, these mechanisms have not yet been well characterized *in vivo* (32, 33). We speculated that the replacement of a cardiac valve is a procedure that could activate the contact pathway and might be involved in the thrombotic or bleeding complications observed in a large proportion of these patients.

In this study, we have analyzed the elements of the contact pathway in patients undergoing two cardiac valve replacement procedures, TAVR or SAVR, with different incidence of thrombotic complications. We showed that these two procedures do not activate the contact pathway, since no activated FXII or kallikrein were observed in any patient by various specific methods.

Two findings were remarkable. First, we identified a patient with congenital FXII deficiency, a rare disorder whose pathogenic relevance remains an enigma. Our data support the null risk of bleeding described for patients with congenital FXII deficiency and animal models (34, 35), as this patient did not suffer bleeding complications during the cardiac valve replacement procedure. Moreover, patient TAVR44 did not suffer any thrombotic complication. This result, despite being from a single patient, is consistent with the proposed antithrombotic role of FXII deficiency (13, 36), and the antithrombotic potential of anti-FXII drugs observed in animal models exposed to blood−contacting medical devices, including catheters, stents, grafts, filters, and extracorporeal organ support systems (ECOS) (6).

Finally, we observed that the procedure reduces the concentration of FXI in the post-procedure sample in a significant proportion of cases. The hypothesis of a consumption of FXI by activation of the contact pathway may be rejected to explain this finding due to the null detection of FXIIa or kallikrein in these samples. FXI consumption by thrombin activation can also be ruled out as our study also finds a minor activation of coagulation by the transcatheter valve replacement regardless the procedure. Further studies are required to unravel the mechanism that leads to this reduction, but it is significantly dependent on the type of procedure. Thus, patients who underwent SAVR showed a greater reduction in FXI after the procedure than patients who underwent TAVR. Independently of the mechanism that explains the differences in FXI levels after valve replacement, it seems that reduction in FXI after the procedure could be considered a good prognostic marker for thrombotic complications, especially when compared with patients with increased FXI after the procedure. These results support the antithrombotic protection associated with reduced FXI levels (9, 10) and the increased risk of thrombosis caused by increased levels of this procoagulant protease (37). However, further studies are required to verify the protection against thrombosis caused by a moderate reduction in FXI levels, since most of the studies showing such protection have been done with more severe reductions of FXI (9, 25, 26, 38).

Our results also highlight the higher frequency of vascular complications in the TAVR procedure. In fact, the most common vascular complications during TAVR are bleeding due to difficult access and closure of the femoral artery. Thrombotic complications in the vascular territory are very rare, except stroke. But stroke seems to be more related to debris produced during valve implantation and to large catheters in the aortic arch (39). The requirement of large vascular introducers used in patients with atheromatous, tortuous and calcified arterial tree due to the older patients selected for this procedure could increase FXI levels by a still unknown mechanism that increases the risk of thrombotic events.

However, the genesis of stroke and other thromboembolic complications during the mid-and long-term follow-up after TAVR and SAVR appears to be multifactorial (40). Although our data suggest minor activation of thrombin generation in these patients, the role of increased platelet activation as main seed of the thromboembolic complications is still to be clarified (41).

In contrast, the evolution of FXI levels after the procedure is not significantly associated with the risk of bleeding. Thus, the incidence of major bleeding was similar in patients who had a reduction in this procoagulant protease than in patients with increased FXI. These results are supported by the minor role of FXI levels on the risk of bleeding, provide new evidence for the safety of FXI-targeted treatments as new antithrombotic therapies (42), and outline a more personalized antithrombotic management strategy.

The main limitation of our study is the relatively small number of patients enrolled. Further studies including a larger patient population are required to validate these results and the potential role of FXI variations in the prognosis of heart valve replacement. Moreover, we restricted our analysis to the pre and post-procedures samples. Studies evaluating the contact pathway in samples collected later would provide valuable information.

## Conclusion

This study shows no relevant activation of the contact pathway of coagulation by TAVR or SAVR. However, the significant reduction of FXI levels that occurs mainly in SAVR, could be a useful biomarker as it associated with lower incidence of thrombotic events. Moreover, these results encourage evaluating the usefulness and safety of antithrombotic treatments targeting FXI in patients underwent these procedures.

## Data availability statement

The original contributions presented in the study are included in the article/Supplementary Material, further inquiries can be directed to the corresponding authors.

## Ethics statement

The studies involving human participants were reviewed and approved by the Hospital Clínico Universitario Virgen de la Arrixaca. The patients/participants provided their written informed consent to participate in this study.

## Author contributions

CL-G, ME-P, EP, VJ-D, JG-L, MG-N, FS-T, SC, PJ-S, and JB-A recruited samples and clinical outcomes. MM-B, AM, and AR performed experimental analysis. MM-B, JC, FM, GM, VJ-D, ME-P, and VV designed the research, evaluate the data, and wrote the manuscript. All authors read and approved the final manuscript.

## Conflict of interest

The authors declare that the research was conducted in the absence of any commercial or financial relationships that could be construed as a potential conflict of interest.

## Publisher’s note

All claims expressed in this article are solely those of the authors and do not necessarily represent those of their affiliated organizations, or those of the publisher, the editors and the reviewers. Any product that may be evaluated in this article, or claim that may be made by its manufacturer, is not guaranteed or endorsed by the publisher.

## Supplementary material

Supplementary Figure 1 Identification of a patient with congenital FXII deficiency who underwent TAVR (TAVR44). The plasma levels of FXII **(A)** and other hepatic proteins, antithrombin (AT) and FXI **(B)** were detected by Western Blot. The sample pre and post-procedure are indicated. As controls we used plasma from a pool of healthy blood donors and from a patient with congenital deficiency of FXII caused by the c.919del G homozygous deletion (FXII congenital deficiency). **(C)** *F12* gene variations identified in patient TAVR44 with congenital FXII deficiency. **(D)** Plasma levels of FXIa in pre- and post-procedure sample from TAVR44 were evaluated by SDS-PAGE Western blot. Plasma from a healthy subject with and without kaolin was used as control.

Supplementary Figure 2 FXI levels (Post/Pre) according to the presence of thrombotic events or periprocedure death. Values are post- respect to pre- procedure FXI (B/A) antigen, %.
