# Supplementary figures and images for: Contact pathway in surgical and transcatheter aortic valve replacement
Source: Front Cardiovasc Med. 2022 Jul 22;9:887664. doi: 10.3389/fcvm.2022.887664 (PMC9354960; doi:10.3389/fcvm.2022.887664)

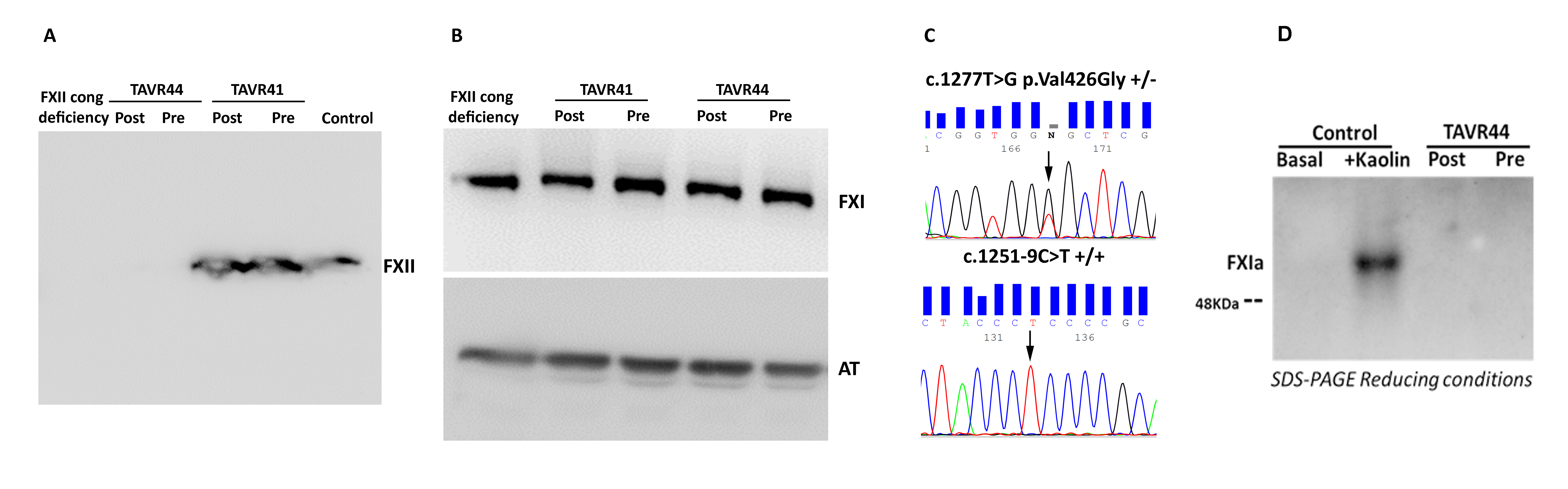

Supplement: Supplementary Figure 1 — Identification of a patient with congenital FXII deficiency who underwent TAVR (TAVR44). The plasma levels of FXII (A) and other hepatic proteins, antithrombin (AT) and FXI (B) were detected by Western Blot. The sample pre and post-procedure are indicated. As controls we used plasma from a pool of healthy blood donors and from a patient with congenital deficiency of FXII caused by the c.919del G homozygous deletion (FXII congenital deficiency). (C) F12 gene variations identified in patient TAVR44 with congenital FXII deficiency. (D) Plasma levels of FXIa in pre- and post-procedure sample from TAVR44 were evaluated by SDS-PAGE Western blot. Plasma from a healthy subject with and without kaolin was used as control. [file Image_1.tif]

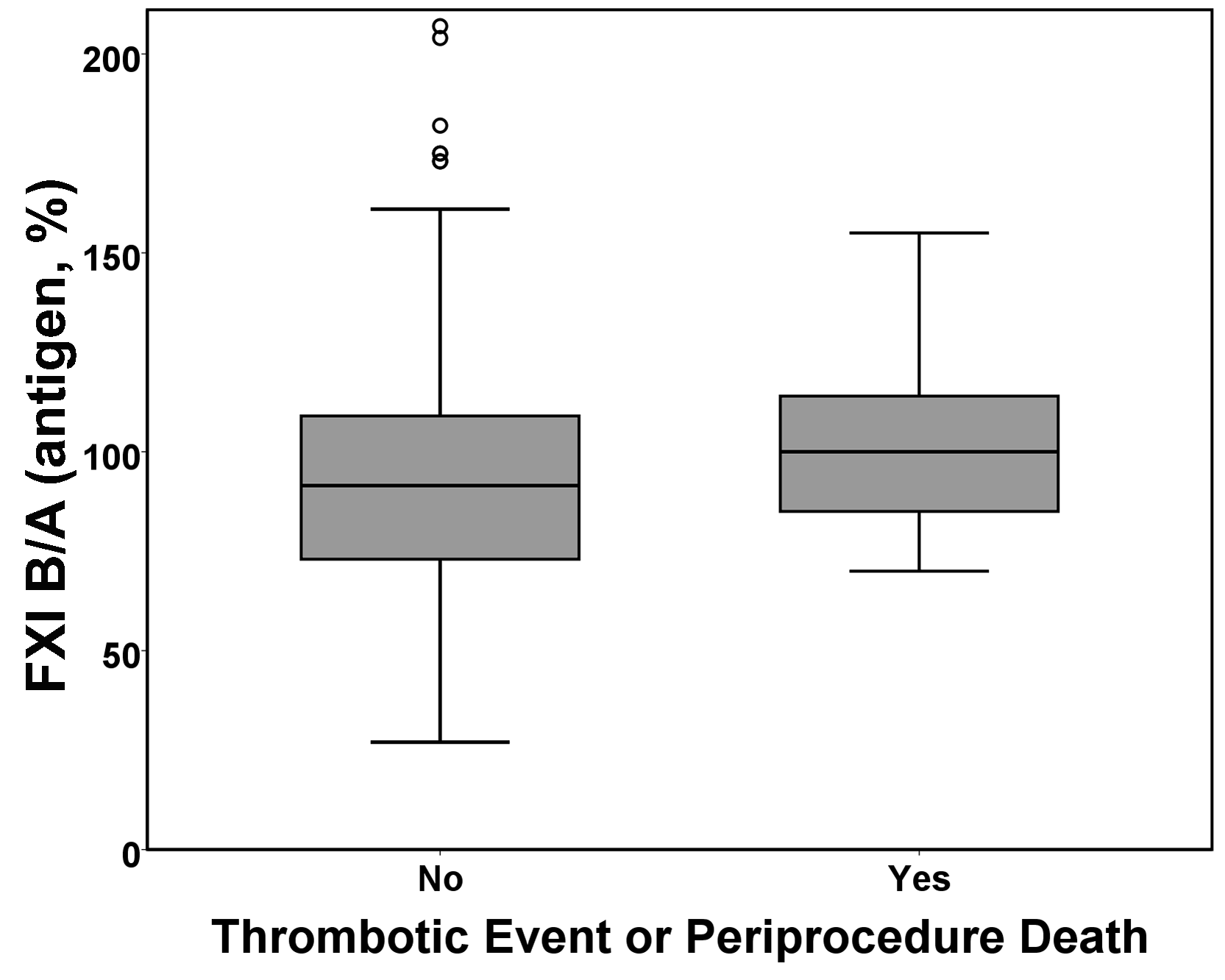

Supplement: Supplementary Figure 2 — FXI levels (Post/Pre) according to the presence of thrombotic events or periprocedure death. Values are post- respect to pre- procedure FXI (B/A) antigen, %. [file Image_2.tif]
